# *Lactiplantibacillus plantarum* N4 ameliorates lipid metabolism and gut microbiota structure in high fat diet-fed rats

**DOI:** 10.3389/fmicb.2024.1390293

**Published:** 2024-06-07

**Authors:** Manqi Deng, Shuaiying Zhang, Siying Wu, Qiunan Jiang, Wenyao Teng, Tao Luo, Yerui Ouyang, Jiantao Liu, Bing Gu

**Affiliations:** ^1^Key Laboratory of Natural Microbial Medicine Research of Jiangxi Province, College of Life Sciences, Jiangxi Science and Technology Normal University, Nanchang, China; ^2^Key Laboratory of Microbial Resources and Metabolism of Nanchang City, College of Life Sciences, Jiangxi Science and Technology Normal University, Nanchang, China

**Keywords:** *Lactiplantibacillus plantarum*, lipid-lowering, gut microbiota, gamma-aminobutyric acid, glutathione metabolism

## Abstract

Lowing blood lipid levels with probiotics has good application prospects. This study aimed to isolate probiotics with hypolipidemic efficacy from homemade na dish and investigate their mechanism of action. *In vitro* experiments were conducted to determine the cholesterol-lowering ability of five isolates, with results showing that *Lactiplantibacillus plantarum* N4 exhibited a high cholesterol-lowering rate of 50.27% and significant resistance to acid (87%), bile salt (51.97%), and pepsin (88.28%) in simulated gastrointestinal fluids, indicating promising application prospects for the use of probiotics in lowering blood lipids. The findings from the *in vivo* experiment demonstrated that the administration of N4 effectively attenuated lipid droplet accumulation and inflammatory cell infiltration in the body weight and liver of hyperlipidemic rats, leading to restoration of liver tissue morphology and structure, as well as improvement in lipid and liver biochemical parameters. 16S analysis indicated that the oral administration of N4 led to significant alterations in the relative abundance of various genera, including *Sutterella*, *Bacteroides*, *Clostridium*, and *Ruminococcus*, in the gut microbiota of hyperlipidemia rats. Additionally, fecal metabolomic analysis identified a total of 78 metabolites following N4 intervention, with carboxylic acids and their derivatives being the predominant compounds detected. The transcriptomic analysis revealed 156 genes with differential expression following N4 intervention, leading to the identification of 171 metabolic pathways through Kyoto Encyclopedia of Genes and Genomes enrichment analysis. Notably, the glutathione metabolism pathway, PPAR signaling pathway, and bile secretion pathway emerged as the primary enrichment pathways. The findings from a comprehensive multi-omics analysis indicate that N4 influences lipid metabolism and diminishes lipid levels in hyperlipidemic rats through modulation of fumaric acid and γ-aminobutyric acid concentrations, as well as glutathione and other metabolic pathways in the intestinal tract, derived from both the gut microbiota and the host liver. This research offers valuable insights into the therapeutic potential of probiotics for managing lipid metabolism disorders and their utilization in the development of functional foods.

## 1 Introduction

Hyperlipidemia constitutes a significant risk factor in the onset of cardiovascular diseases (Stewart et al., 2020). Over recent decades, a plethora of synthetic drugs have emerged for hyperlipidemia management. Nevertheless, clinical evidence supports the occurrence of specific adverse effects associated with most lipid-lowering drugs (Dybiec et al., 2023). Recent studies have underscored the lipid-lowering effects of probiotics. Probiotics modulate lipid metabolism via two key metabolites, short-chain fatty acids (SCFAs) and secondary bile acids (BAs). BAs, pivotal signaling molecules, significantly contribute to fat digestion by emulsifying fats into smaller particles, subsequently broken down into fatty acids by lipoprotein lipase (Yang et al., 2022). SCFAs, beneficial metabolites resulting from probiotic fermentation, primarily encompass acetic, propionic, and butyric acids, which also play an important role in inhibiting cholesterol synthesis. For instance, short-chain fatty acids derived from *Lactobacillus* strains inhibit cholesterol synthesis by attenuating the activity of DL-3-hydroxy-3-methylglutaryl coenzyme A (HMG-COA) reductase (Tian et al., 2022). Furthermore, research indicates that SCFAs and BAs prompt the secretion of gut hormones implicated in lipid metabolism (Chen et al., 2023). Moreover, certain enzymes and inhibitors generated by probiotics modulate the body’s lipid metabolism, consequently lowering cholesterol levels. For instance, *Lactobacillus* produces cholesterol dehydrogenase/isomerase, which transforms cholesterol into coprosterol, subsequently expelled from the body through feces (Singhal et al., 2019). Through binding to and stimulating hepatic X receptors, *Lactobacillus* effectively downregulates host cholesterol synthesis (Ye et al., 2022). Additionally, *Lactobacillus* induces activation of the Farnesoid X receptor, leading to the downregulation of the expression of key enzymes in bile acid synthesis, namely cholesterol-7α-hydroxylase (CYP7A1) and sterol-12α-hydroxylase (CYP8B1). This modulation ultimately results in decreased cholesterol levels *in vivo* (Lim et al., 2020). Furthermore, the secretion of bile salt hydrolase (BSH) by *Lactobacillus* spp. contributes to the reduction in host cholesterol levels by hydrolyzing bound bile acids within the organism, thereby preventing their absorption (Hernández-Gómez et al., 2021).

The liver serves as the primary organ for lipid metabolism within the body, functioning as a central hub for various physiological processes. It plays crucial roles in nutrient metabolism, supporting the immune system, controlling endocrine signaling pathways related to growth, maintaining lipid and cholesterol homeostasis, and serving as a key site for glutathione (GSH) metabolism (Trefts et al., 2017). Among these functions, GSH and its related enzymes form an antioxidant defense system that plays a key role in protecting hepatocytes from oxidative stress and other potential injuries (Liu et al., 2021). GSH is a tripeptide containing a γ-amide bond and sulfhydryl group, consisting of glutamic acid, cysteine and glycine. It is an ubiquitous mammalian antioxidant that plays an important role in resistance to oxidative damage, nutrient metabolism, and numerous regulatory cellular events, including gene expression, DNA and protein synthesis, cell proliferation and apoptosis, iron death, signal transduction, cytokine production and immune responses, and protein glutathionylation (Wang et al., 2021; Zhang et al., 2021). Simultaneously, the liver plays a central role in REDOX reactions, thereby contributing substantially to metabolic regulation and safeguarding. Oxidative stress additionally plays a role in the functionality of beige adipose tissue, where adipocytes can chelate succinic acid and oxidize it in the mitochondria, promoting thermogenic respiration (Fuentes et al., 2013). Studies have indicated that specific anti-cancer medications, polysaccharides, and isolated compounds can influence lipid metabolism disorders and gut microbiota composition in rats consuming high-fat diets, primarily through REDOX reactions (Guo et al., 2018; Hu et al., 2021). Recent research indicates that the gut microbiota plays a significant role in modulating the body’s metabolism, thereby influencing the development and advancement of chronic conditions like obesity, diabetes, and atherosclerosis (Hasani et al., 2021). While the hypolipidemic effects of certain probiotic strains are established, further research is needed to fully understand the mechanisms by which probiotics regulate lipid metabolism, as various strains may operate through distinct pathways.

Na dish is a traditional local dish from Jiangxi Province in China, which is a plant-based natural fermented food. The primary objective of this research was to isolate potential probiotic strains with excellent cholesterol-lowering capabilities from na dish, particularly *Lpb. plantarum* N4, and explore its potential therapeutic advantages in alleviating hyperlipidemia. This study entailed evaluating alterations in intestinal microbiota, liver transcriptome, and fecal metabolites to uncover the mechanisms at play. By examining the impact of *Lpb. plantarum* N4 consumption on intestinal microorganisms and establishing correlations between fecal metabolite profiles and intestinal microbiota, the study aimed to lay the groundwork for developing functional *Lactobacillus*-based products for the prevention of hyperlipidemia.

## 2 Materials and methods

### 2.1 Isolation and purification of lactic acid bacteria from na dish

Lactic acid bacteria were isolated using a previously established method with minor modifications (Edith Marius et al., 2018). Weigh 1 g of na dish and add 9 mL of saline solution in a centrifuge tube, then mix thoroughly on a shaker. Take 1 mL of the suspension and dilute it with saline solution to 10^−3^ using a saline gradient in a new centrifuge tube. Take 100 μL of the 10^−3^ dilution and spread it evenly on an MRS agar plate containing calcium carbonate. Incubate the plate at 37°C for 24 h. Based on the acid-producing properties of lactic acid bacteria, a calcium-soluble spherical shape will appear around the colony. The colony morphology of lactic acid bacteria is typically smooth-edged, round, white or lightly yellow colonies. Therefore, select colonies with calcium-soluble spherical shape and smooth-edged, white or light yellow, round morphology as potential candidate strains. This colony was then subjected to further cultivation in MRS broth at 37°C for 24 h and subsequently purified by re-streaking on MRS agar. The reference type strain used was *Lpb. plantarum ATCC 14917*, obtained from BeNa Culture Collection Co., Ltd. (Beijing, China).

### 2.2 Cholesterol-lowering activity of strains

The OPA method was used to quantify the cholesterol contents of the samples (Wang et al., 2019). Bacterial cultures in the logarithmic growth phase (1%, v/v) were introduced into MRS broth containing cholesterol and incubated at 37°C for 24 h under anaerobic conditions. After centrifugation at 5,000 rpm for 10 min, the supernatant was collected and combined with 3 mL 95% ethanol and 2 mL KOH. This mixture was heated at 60°C for 30 min. After cooling, 3 mL n-hexane and 3 mL distilled water were added, and the mixture was dried at 60°C. Following the addition of 2 mL o-phthalaldehyde reagent and concentrated sulfuric acid, the mixture was stirred and left undisturbed for an additional 10 min. The absorbance at 550 nm was measured for both the uninoculated and inoculated samples.

### 2.3 Identification of isolates through sequencing the 16S rDNA

The method outlined by Sui et al. was used for PCR amplification of the 16S rDNA of the selected strains (Sui et al., 2021). PCR amplification of 16S rDNA from a bacterial colony of the isolate was carried out using the 27F primer (5’-AGAGTTTGATCCTGGTCAG-3′) and 1492R (5’-GGTTACCTTGTTACGACTT-3′) prime. Subsequently, the amplified segments were sent to a commercial enterprise in Shanghai, China for DNA sequencing. The Basic Local Alignment Search Tool^1^ was used to analyze the sequence fragments by referencing the GenBank DNA database. A phylogenetic tree was constructed to identify the most closely related bacterial species using MEGA 7 software.

### 2.4 Acid tolerance tests

Acid resistance was evaluated by the viable plate count method (Nishida et al., 2008). Bacteria cultured in MRS liquid medium at 37°C until they reached logarithmic phase were harvested by centrifugation (10,000 rpm for 5 min). To achieve an OD600 of 0.5, the collected cells were diluted with phosphate-buffered saline (PBS, pH 3.0). Viable counts were determined on MRS agar plates after 0 and 3 h of incubation at 37°C, this process was repeated three times. Survival rate was calculated using the following equation:


$$\begin{array}{l} \mathrm{Survival}\;\mathrm{rate}\;\left(\%\right)=\left(CFU\;\mathrm{of}\;\mathrm{final}\;\mathrm{viable}\;\mathrm{cells}\;\mathrm{inoculated}\right)/\\ \;\left(CFU\;\mathrm{of}\;\mathrm{initial}\;\mathrm{viable}\;\mathrm{cells}\;\mathrm{inoculated}\right)\times 100. \end{array}$$

### 2.5 Bile tolerance tests

To evaluate the resilience of the isolates to bile salt conditions, the viable plate count technique was employed (Azat et al., 2016). The bacteria were cultivated at 37°C until they reached the exponential growth phase, at which point they were harvested by centrifugation at 10,000 rpm for 5 min. Subsequently, the collected cells were adjusted to an OD600 of 0.5 using PBS with a pH of 7.0. The cells were then plated on MRS broth containing 0.3% bile salts. Viable counts were determined on MRS agar plates after 0 and 3 h of incubation at 37°C. The previously mentioned equation was used to calculate the survival rate.

### 2.6 Tolerance to simulated gastrointestinal conditions

To evaluate the resilience of the isolates to simulated gastrointestinal transport conditions, the viable plate count technique was employed (Tokatlı et al., 2015). In brief, artificial gastric fluid was prepared by mixing 3.5 g/L pepsin (Sigma-Aldrich, South Korea) with a sterile saline solution and adjusting its acidity to pH 3 using HCl. Bacterial cultures were inoculated at a concentration of 1% (v/v) and cultivated at 37°C until they reached logarithmic growth phase. Subsequently, the cells were harvested by centrifugation at 10,000 rpm for 5 min. The collected cells were diluted using PBS (pH 7.0 until they reached an OD600 of 0.5). Viable counts were determined on MRS agar plates after 0 and 3 h of incubation at 37°C. The previously mentioned equation was used to calculate the survival rate.

### 2.7 Bile salt hydrolase activity

The bile salt hydrolase (BSH) functions of the probiotic strains were assessed according to the procedure described by Choi and Chang (2015). Overnight cultures were spread on MRS agar medium supplemented with 0.5% sodium taurine deoxycholic acid and 0.37 g/L CaCl_2_. The plates were placed in an anaerobic environment and incubated at 37°C for 48 h. The presence of a white precipitate surrounding the filter paper confirms the presence of BSH within the precipitation circle.

### 2.8 *In vitro* safety assessment

#### 2.8.1 Hemolytic activity

The hemolysis of lactic acid bacteria was assessed as described previously (Palaniyandi et al., 2017). A single strain was examined using Columbia blood agar (BD, Difco). After a 2-day incubation period at 37°C, a thorough analysis was conducted to determine the presence of either α- or β-hemolysis following colony growth.

#### 2.8.2 Determination of antibiotic susceptibility

Sensitivity to each antibiotic was determined according to the guidelines established by the European Food Safety Authority (Yadav et al., 2016). Ten antibiotics (all sourced from Sigma-Aldrich) were used to evaluate susceptibility to a range of antibiotics including cefazolin (30 μg), chloramphenicol (30 μg), azithromycin (15 μg), gentamicin (20 μg), ampicillin (20 μg), amoxicillin (10 μg), vancomycin (30 μg), tetracycline (30 μg), imipenem (10 μg), and clindamycin (2 μg). For this assessment, 50 μL of fresh culture was spread evenly on an MRS agar plate. Subsequently, paper disks, each with a 6 mm diameter and containing the respective antibiotics, were placed on the MRS AGAR plate. After 48 h incubation period at 37°C, a caliper was used to precisely measure the dimensions of the inhibition zones with an accuracy of 0.02 mm.

### 2.9 Animal experiment

#### 2.9.1 Animals and diets – animal model building

Animal experiments were conducted in accordance with internationally recognized guiding principles and protocols, following approval by the Animal Ethics Committee of Jiangxi Science and Technology Normal University. Thirty-two SD rats, aged 5 weeks and weighing approximately 170 ± 5 g, were used for the study. The rats were housed in well-ventilated chambers at a constant temperature of 22 ± 2°C and a humidity level of approximately 60% ± 5%. After a one-week acclimatization period, 32 rats were randomly divided into two groups: a control group (8 rats) and a model group (24 rats). Throughout the study, the rats’ body weights were monitored, and their levels of total cholesterol (TC), triglycerides (TG), low-density lipoprotein (LDL), and high-density lipoprotein (HDL) were measured at weekly intervals. Statistical analysis revealed that compared to the control group, TC, TG, and LDL in model group were significantly increased, while HDL was significantly decreased (*p* < 0.05), confirming the successful establishment of the hyperlipidemic rat model. Subsequently, the rats were further randomized into four treatment groups, each consisting of eight rats. The control group (Control) received a standard fat diet. The model group (HFD) was administered the same quantity of normal saline via a high-fat diet. The experimental group (HFD + N4) was fed a high-fat diet and supplemented with N4 (5 × 10^9^ CFU/rat per day). The N4 cells was collected and dissolved in normal saline, with a bacterial concentration of 5 × 10^9^ CFU/mL. The rats were administered the N4 suspension by gavage, with each rat receiving 3 mL per day. The treatment group (HFD + Ator) received a high-fat diet with atorvastatin calcium trihydrate at a daily dose of 4.07 mg/kg. All diets were sourced from Shenyang Maohua Biotechnology Products (Liaoning, China). The body weight of each rat was recorded weekly for a total of 8 weeks before euthanization.

#### 2.9.2 Collection and preparation of samples

In the 8th week of the experiment, fecal samples were collected from the rats. Subsequently, these specimens were rapidly frozen in liquid nitrogen and preserved at −80°C. Each rat was administered ketamine (100 mg/kg BW) to induce anesthesia and subsequently euthanized for anatomical examination. The obtained serum and liver tissue samples were quickly frozen in liquid nitrogen for 30 s and then maintained at −80°C until subsequent analysis.

#### 2.9.3 Analysis of the serum samples using biochemical assays

Serum levels of TC, TG, HDL-C, and LDL-C were determined using biochemical kits sourced from the Nanjing Jiancheng Bioengineering Institute, Nanjing, China. These experiments were conducted in strict accordance with the manufacturer s instructions.

#### 2.9.4 Liver tissue histological examination

Liver samples were preserved overnight in 4% paraformaldehyde (Biochem, China) at 4°C. Subsequently, they were sectioned into slices with a thickness of five micrometers using the Leica RM2235 microtome from Heidelberg, Germany. The sections were then subjected to hematoxylin and eosin staining. To visualize the stained liver sections, an optical microscope (Olympus, Tokyo, Japan) equipped with a digital camera was used.

#### 2.9.5 High-throughput sequencing analysis of the gut microbiota

Considering the variations among rats within each group, including factors such as weight, serum levels, and liver biochemical parameters, six rats from each group were selected for high-throughput sequencing analysis of the gut microbiota. DNA extraction from fecal samples was performed using a Fast DNA SPIN Extraction Kit (MP Biomedicals, Santa Ana, CA, United States). To amplify the V3-V4 regions of the 16S rRNA gene, forward primer 27F (5’-ACTCCTACGGGAGGCAGCA-3′) and reverse primer 1492R (5’-GGACTACHVGGGTWTCTAAT-3′) were used. Sequencing of PCR products was performed at Personal Biotechnology Co., Ltd. (Shanghai, China) using the Illumina platform, with a focus on achieving high-throughput sequencing. These sequences were classified into operational taxonomic units (OTUs) at a threshold of 97% using UCLUST. Alpha-diversity metrics and principal coordinates analysis, were calculated and visualized using R software (v3.3.3). Significant distinctive OTUs were identified using linear discriminant analysis effect size (LEfSe) analysis with a significance level set at 0.05.

#### 2.9.6 Metabolomics of fecal samples using UPLC-QTOF/MS

After excluding rats with significant individual differences within each group, six rats were selected from each group for fecal metabolomic analysis. A 25 mg fecal sample was combined with an extract composed of acetonitrile, methanol, and water in a ratio of 2:2:1 (1,000 μL). The mixture was then homogenized, subjected to ultrasonic processing, and centrifuged. The supernatant (800 μL) was collected in a new test tube and dried under vacuum at 37°C. After drying, the sample was reconstituted in 200 μL 50% acetonitrile and centrifuged for 10 min. The liquid above the sediment was then transferred to a new glass container for UPLC-QTOF/MS analysis. Additionally, a quality control sample was prepared by combining equal portions of the treatment supernatants from each sample.

The separation of UPLC was carried out using an Agilent Technologies 1,290 Infinity Series UPLC system with a UPLC BEH amide column (2.1 × 100 mm, 1.7 μm, Waters). The mobile phase consisted of aqueous solutions of ammonium acetate, ammonium hydroxide (A), and acetonitrile (B) at a concentration of 25 mmol/L. The elution gradients used for analysis were as follows: 00.5 min, 95% B; 0.57.0 min, 95%65% B; 7.0–8.0 min, 65–40% B; 8.0–9.0 min, 40% B; 9.0–9.1 min, 40%-95 B; 9.112.0 min, B. The column temperature was maintained at 25°C, while the automatic sampler temperature was set at 4°C with a sample size of 2 μL. The UPLC-QTOF/MS data were processed using the MassLynx 4.1 software (Waters Co., United States) for peak detection, filtering, denoising, alignment, and standardization. The calculated data, including retention times (RT), *m*/*z* values and corresponding peak intensity were imported into the R software package for principal component analysis (PCA), partial least squares discriminant analysis (PLS-DA), and orthogonal partial least squares discriminant analysis (OPLS-DA) analyses. To identify potential biomarkers, the variable importance in projection (VIP) value and *p*-value obtained from the two-tailed t-test were utilized. Biomarker identification involved comparing the accurate mass and typical MS/MS fragments with the Human Metabolome Database.^2^ KEGG enrichment analysis was performed using “clusterprofiler”, and the hypergeometric distribution method to calculate the *p*-value (the criterion for significant enrichment was *p*-value <0.05). Additionally, information derived from the KEGG database available on MetaboAnalyst’s website^3^ was used for further analysis.

#### 2.9.7 Analysis of RNA-sequencing in the liver

Eukaryotic reference transcriptome assays were performed for transcriptome sequencing. Clean data were obtained from the raw data by removing reads containing adapters and low-quality reads using the FASTX-Toolkit.^4^ The clean reads were aligned to the reference gene using Bowtie2 software, while the reference genome was mapped using HISAT software. PCA and differential expression analysis of genes (DEGs) were conducted using R package models^5^ to elucidate the structures and relationships among the samples. These DEGs were further analyzed for the enrichment of Gene Ontology functions and KEGG pathways. Distinguishing genes were determined by considering the absolute value of the log2-fold change in gene expression levels to be less than 1 and a significance *p*-value less than 0.05.

#### 2.9.8 The real-time qPCR analysis

Total RNAs was extracted from liver tissues using the RNAiso Plus kit (Takara, Japan). Subsequently, cDNA synthesis was performed using the PrimeScript™ RT Kit (Takara, Code No. RR047A). To validate the RNA-seq results, co-expressed genes with varying expression levels were further investigated using the CFX96 RT-PCR Detection System (AB7300, Applied Biosystems, United States) and SYBR^®^ Premix Ex Taq™ II (Takara, Code No. RR820A, Dalian, China). The mRNA levels of each gene were normalized to the geometric mean of β-actin. The primer sequences used for qPCR are listed in Supplementary Table S1.

### 2.10 Result analysis

Data are presented as mean ± standard deviation (SD). Statistical analysis of the test results was performed using GraphPad Prism 8.0, employing one-way analysis of variance (ANOVA), followed by Tukey’s multiple comparison test. The test results were considered statistically significant at *p* < 0.05, and high significance levels were set at *p* < 0.01.

## 3 Results

### 3.1 Isolated strain with cholesterol-lowering effect – *Lactiplantibacillus plantarum* N4

Five strains with cholesterol-lowering efficacy were isolated from traditional fermented na dish (Table 1). The colonies typically appeared circular, white, had a smooth texture, and appeared moist. Gram staining revealed purple cells under a microscope, classifying them as gram-positive bacteria (G^+^). Among these strains, N4 strain exhibited the highest cholesterol degradation rate of 50.27%. Furthermore, 16S rRNA sequencing revealed that the 16S rRNA genetic sequence of N4 exhibited over 95% similarity to that associated with *Lpb. plantarum* (Supplementary Figure S1). Therefore, strain N4 was assigned to species *Lpb. plantarum*.

**Table 1:** Cholesterol degradation rate of selected strains from na dish.

Strain	Cholesterol degradation rate (%)
N1	44.95 ± 3.27^***^
N2	48.29 ± 1.00^***^
N3	26.73 ± 2.09
N4	50.27 ± 2.38^***^
N5	43.96 ± 2.40^***^
Control	30.00 ± 1.00

Values are expressed in mean ± standard deviation (*n* = 3). Control: Lpb. plantarum ATCC 14917. Comparison of the Lpb. plantarum ATCC 14917 strain with other strains: *p < 0.05, **p < 0.01, ***p < 0.001.

### 3.2 *Lactiplantibacillus plantarum* N4 exhibits a favorable survival rate under simulated gastrointestinal conditions

The strain N4 exhibited robust tolerance to acidic environments and simulated gastric juices, with an impressive survival rate of 87% under acidic conditions, indicating resistance to low pH levels (Table 2). Furthermore, it displayed considerable resilience, maintaining a survival rate of 51.97% even after 3 h exposure to 0.3% bile salts. Following exposure to both acidic conditions and pepsin, the strains exhibited commendable survivability, with an 88.28% survival rate observed under simulated gastric fluid conditions.

**Table 2:** Detection of strain *Lpb. plantarum* N4 acid tolerance, bile tolerance, and pepsin tolerance.

Gastrointestinal transport condition	N4 Survival rate (%)	Control Survival rate (%)
Acid tolerance	87.00 ± 6.18	89.87 ± 7.16
Bile tolerance	51.97 ± 8.04^**^	77.00 ± 2.00
Pepsin tolerance	88.28 ± 0.78^**^	82.00 ± 2.00

Values are expressed in mean ± standard deviation (*n* = 3). Control: Lpb. plantarum ATCC 14917. Comparison of the Lpb. plantarum ATCC 14917 strain with other strains: *p < 0.05, **p < 0.01, ***p < 0.001.

### 3.3 *Lactiplantibacillus plantarum* N4 possesses BSH enzyme activity and safety

BSH enzyme activity has become a crucial indicator for *in vitro* screening of cholesterol-lowering lactic acid bacteria. The results clearly revealed distinct white precipitate rings surrounding the filter paper of strain N4, providing strong evidence of potentially robust BSH enzyme activity (Supplementary Figure S2). The N4 strain exhibited no hemolytic activity, as demonstrated in Supplementary Figure S3. Antibiotic sensitivity was evaluated using the paper diffusion method to determine the inhibitory bands of 10 antibiotics. According to these findings (Table 3), the N4 strain displayed minimal susceptibility to most antibiotics, with the exception of vancomycin.

**Table 3:** Susceptibility of strain *Lpb. plantarum N4* to ten antibiotics.

Antibiotic	Concentration (μg/disk)	N4	Control
gentamicin	20 μg	S	S
chloramphenicol	30 μg	S	S
imipenem	10 μg	S	S
azithromycin	15 μg	S	S
vancomycin	30 μg	--	--
clindamycin	2 μg	S	I
cefazolin	30 μg	R	S
amoxicillin	10 μg	S	S
tetracycline	30 μg	S	S
ampicillin	20 μg	S	S

R, resistant; S, sensitive; I, moderately sensitive; −, no sensitivity. Control: Lpb. plantarum ATCC 14917.

### 3.4 *Lactiplantibacillus plantarum* N4 reduces body weight and lipid levels in hyperlipidemic rats

**Figure 1:**
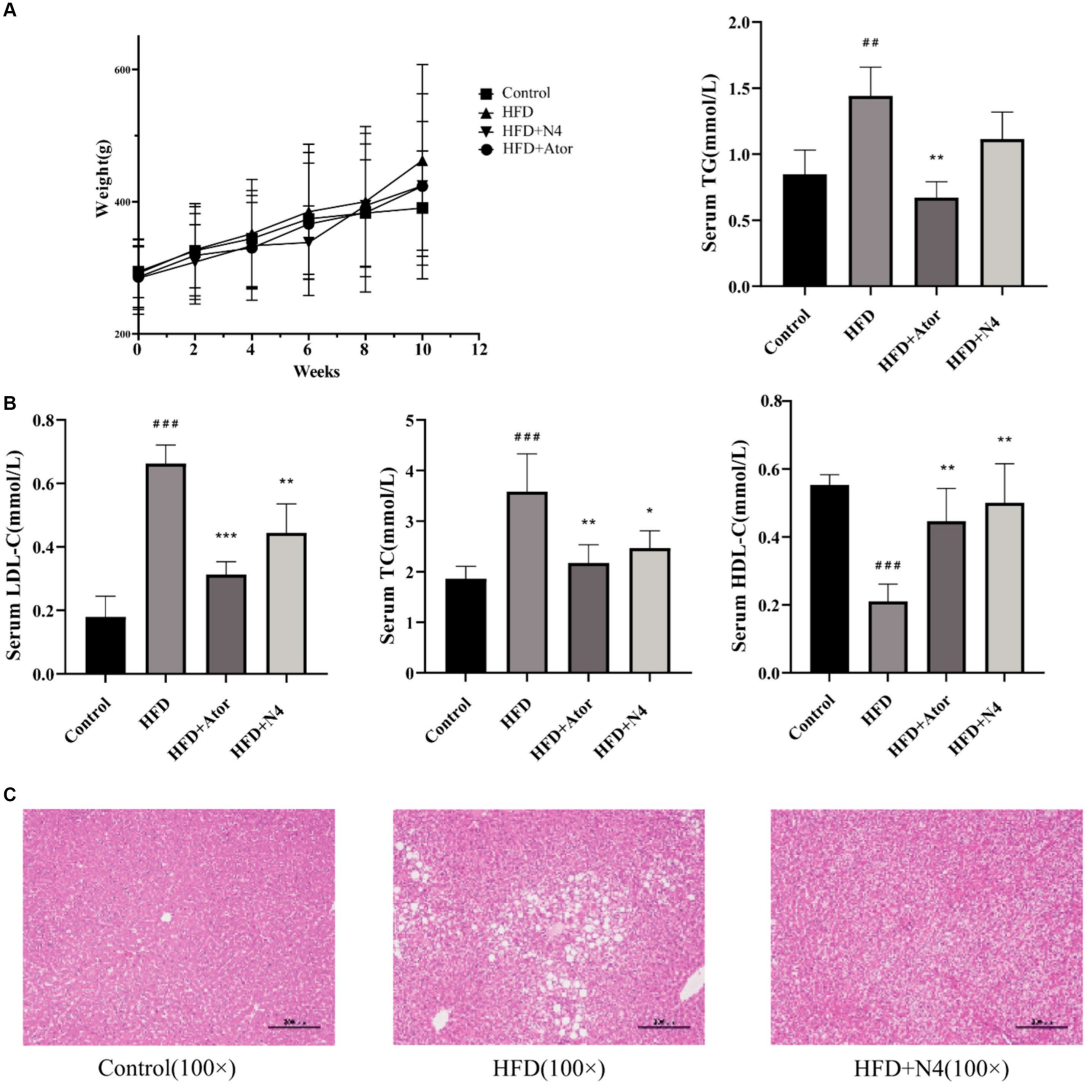
The biochemical indicators in serum and liver tissue. **(A)** Body weight evolution during the experimental period of four groups of rats. **(B)** Effects of N4 administration on the serum biochemical parameters (TC, TG, HDL-C, and LDL-C) in rats fed a high-fat diet for consecutive 8 weeks. **(C)** Histological changes of liver sections were measured by HE staining at 100× magnification, and observed under a light microscope. The results are presented as mean ± SD (n = 6/group). *p < 0.05, **p < 0.01, and *** *p* < 0.001 as compared with the HFD group. ^#^*p* < 0.05, ^##^*p* < 0.01, and ^###^*p* < 0.001 as compared with the Control group. One-way analysis of variance was used to test the significance difference between groups. TG, triglyceride; LDL-C, low-density lipoprotein cholesterol; HDL-C, high-density lipoprotein cholesterol. Control, normal-fat diet group; HFD, high-fat diet group; HFD + Ator, high-fat diet group treated with atorvastatin calcium tablet; HFD + N4, high-fat diet group treated with *Lpb. plantarum N4.*

At the end of the trial, there was a trend of a decrease in body weight in the HDF + N4 group compared to the HFD group, but it was not statistically significant (Figure 1A). After an 8-week intervention, the incorporation of N4 into the diet significantly reduced the concentrations of serum TC and LDL-C in rats fed a high-fat diet (*p* < 0.05). Furthermore, it significantly elevated serum HDL-C levels (*p* < 0.01). However, there was no significant decrease in serum TG concentrations. Administering Atorvastatin at a dose of 15 mg/kg resulted in a significant reduction in serum TG, TC, and LDL-C levels and an increase in serum HDL-C levels (*p* < 0.01). The results showed that *L. plantarum* N4 significantly decreased the blood lipid levels of rats in the HFD group (Figure 1B).

### 3.5 *Lactiplantibacillus plantarum* N4 inhibits hepatic lipid accumulation in hyperlipidemic rats

Figure 1C demonstrates that histological analysis of the liver through hematoxylin–eosin staining indicated the presence of lipid accumulation and steatosis in the hepatocytes of rats subjected to a high-fat diet. Conversely, the control group exhibited livers devoid of these pathological conditions. Nevertheless, after an 8-week intervention with N4, there was a notable reduction in hepatic lipid droplet accumulation attributed to the high-fat diet. These results imply a significant amelioration of hepatic steatosis induced by a high-fat diet in the treatment group.

### 3.6 *Lactiplantibacillus plantarum* N4 improves intestinal microbiota diversity in hyperlipidemic rats

**Figure 2:**
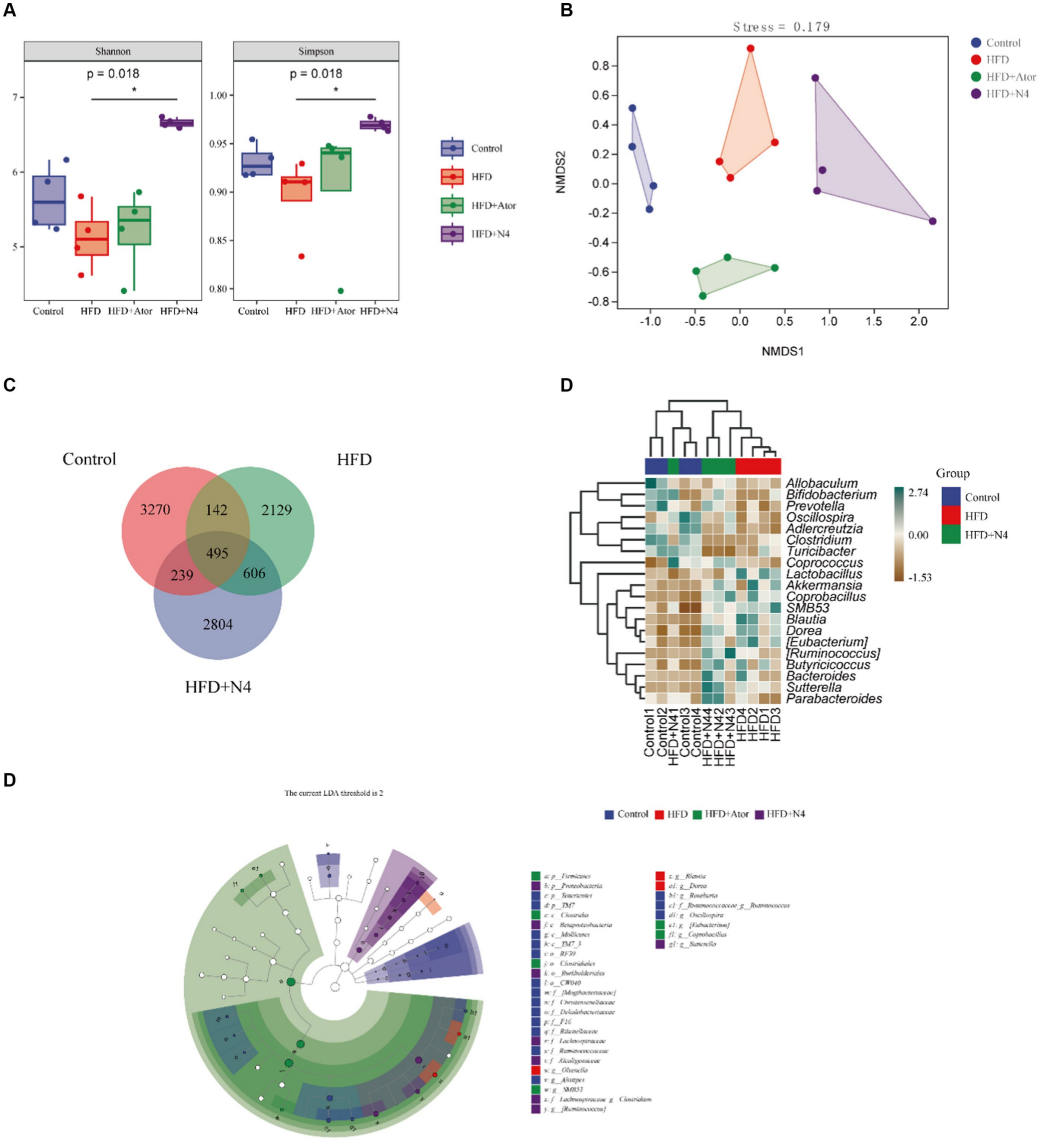
Effects of N4 on gut microbiota based on 16S rRNA sequencing. **(A)** Grouping box graph of the Alpha diversity index. **(B)** NMDS cluster analysis diagram. **(C)** Venn Diagram. **(D)** Heat map of species composition. **(E)** taxonomic cladistics of LEfSe analysis. Data are shown as mean ± SD (*n* = 6/group). One-way analysis of variance was used to test the significance difference between groups. Control, normal-fat diet group; HFD, high-fat diet group; HFD + Ator, high-fat diet group treated with atorvastatin calcium tablet; HFD + N4, high-fat diet group treated with *Lpb. plantarum N4.*

In Figure 2A, the Shannon index and Simpson index were elevated in the HFD + N4 group compared to the HFD group (*p* < 0.05), suggesting that supplementation with *Lpb. plantarum* N4 restored the microbial abundance in their intestinal tract. According to Figure 2B of NMDS cluster analysis, the four sample groups exhibited non-overlapping and were completely separated. According to the Venn diagram (Figure 2C), there were a total of 606 OTU sequences in the HFD and HFD + N4 groups. To further compare the differences in species composition between samples, we used the R language to create heat maps of species composition (Figure 2D). The results showed that the dominant genera in the HFD group were *Lactobacillus*, *Blautia*, and *Bifidobacterium*, whereas after treatment, the dominant genera in the HFD + N4 group changed to *Sutterella*, *Bacteroides*, and *Ruminococcus*, which predicted them as possible biomarkers. Afterward, we employed LEfSe analysis with an LDA threshold set to 4 to identify potential biomarkers of the gut microbiota within all groups (Figure 2E). We confirmed *Clostridium* and *Ruminococcus* as key intestinal microbiota in the HFD + N4 group, serving as biomarkers.

### 3.7 *Lactiplantibacillus plantarum* N4 elevates carboxylic acid and its derivatives in the feces of hyperlipidemic rats

**Figure 3:**
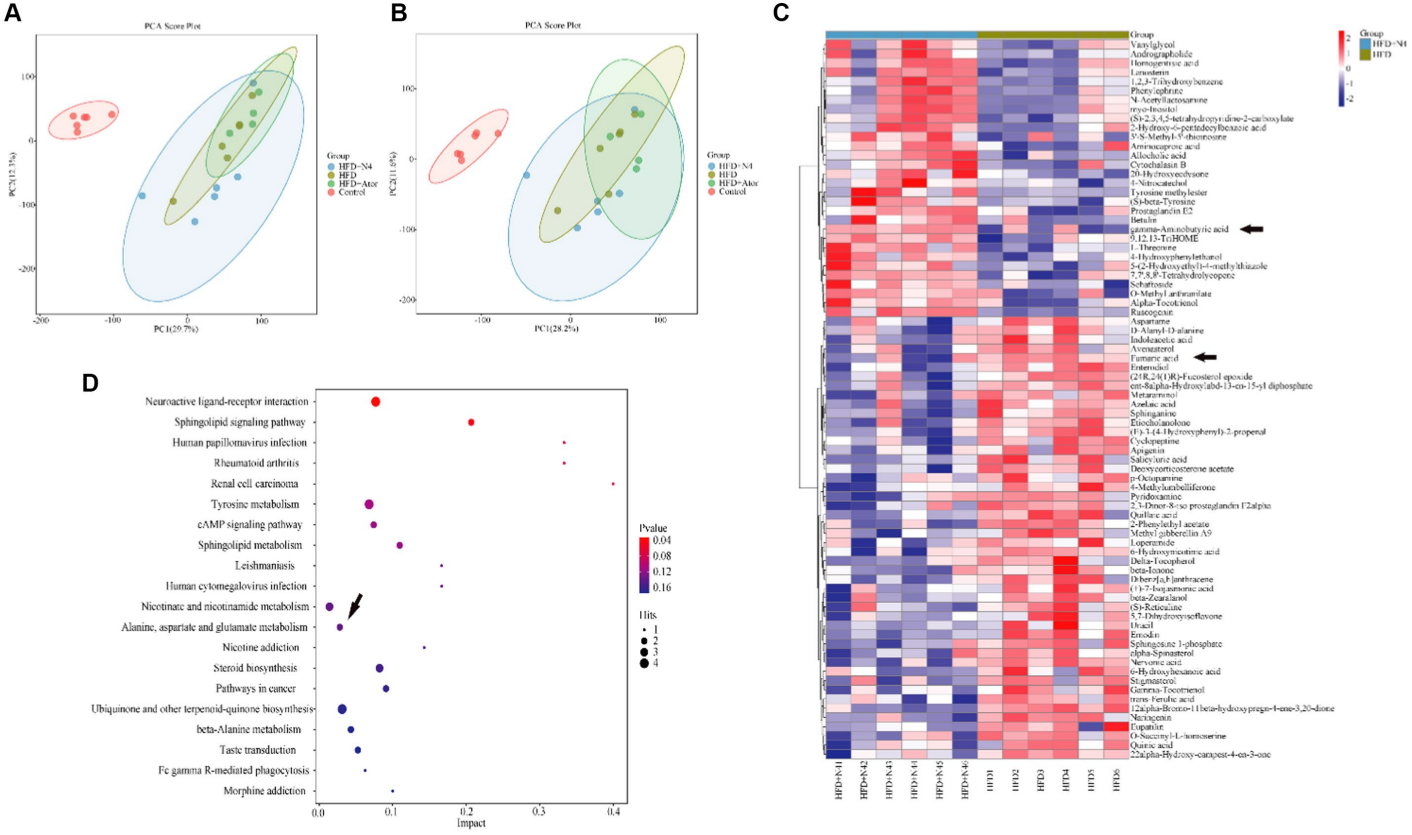
Effect of N4 intervention on metabolome of rat fecal. OPLS-DA score plot of HFD + N4 group and HFD group **(A)** in the positive ion mode and **(B)** in the negative ion mode. **(C)** The heatmap of the relative abundance of significant different metabolites (VIP > 1.0, and *p* < 0.05) in the HFD and HFD + N4 groups. **(D)** The metabolic pathway impact prediction between HFD and HFD + N4 groups in liver tissues based on KEGG online database. Data are shown as mean ± SD (*n* = 6/group). One-way analysis of variance was used to test the significance difference between groups. Control, normal-fat diet group; HFD, high-fat diet group; HFD + Ator, high-fat diet group treated with atorvastatin calcium tablet; HFD + N4, high-fat diet group treated with *Lpb. plantarum N4.*

The results of the PCA analysis did not effectively differentiate the intergroup differences among the four sample groups (Supplementary Figures S4A,B). However, the subsequent PLS-DA analysis demonstrated a significant improvement in distinguishing these differences (Supplementary Figures S4C,D). Furthermore, an additional OPLS-DA analysis was performed specifically for the HFD and N4 groups. Interestingly, the samples from these two groups exhibited a clear separation in both positive and negative ion modes (Figures 3A,B). This separation strongly demonstrated that high-fat diet-fed rats showed significant alterations in fecal metabolites after N4 supplementation. A total of 78 potential biomarkers successfully discovered when comparing the HFD and HFD + N4 groups (Figure 3C). Among these, 30 metabolites exhibited a substantial increase, whereas 48 metabolites displayed a notable decrease in regulation. These metabolites, spanning various chemical classes such as benzene and its substituted derivatives, carboxylic acids and their derivatives, prenol lipids, steroids and steroid derivatives, and others, were differentially expressed. Of particular interest among them, Fumaric acid showed a significant downward adjustment and gamma-Aminobutyric acid (GABA) showed a significant upward adjustment. Both belong to carboxylic acids and their derivatives.

KEGG analysis was conducted on 78 significantly different metabolites, and the top 20 enriched pathways were selected for display in a bubble plot (significance threshold set at *p* < 0.05). The KEGG (Figure 3D) analysis revealed several noteworthy pathways related to lipid metabolism. These pathways include interactions between neuroactive ligands and receptors, signaling pathways involving sphingolipids, tyrosine-related signaling pathways, cAMP-related signaling pathways, sphingolipid metabolism, alanine, aspartate, and glutamate metabolism, and sterol biosynthesis. It is worth noting that these metabolic pathways are associated with either Fumaric acid or GABA, but rather both happen to be differential metabolites of the alanine, aspartate, and glutamate metabolism at the same time. In addition, this pathway was also a common metabolic pathway in the transcriptome KEGG enrichment analysis.

### 3.8 *Lactiplantibacillus plantarum* N4 modulates hepatic glutathione metabolic pathways in hyperlipidemic rats

**Figure 4:**
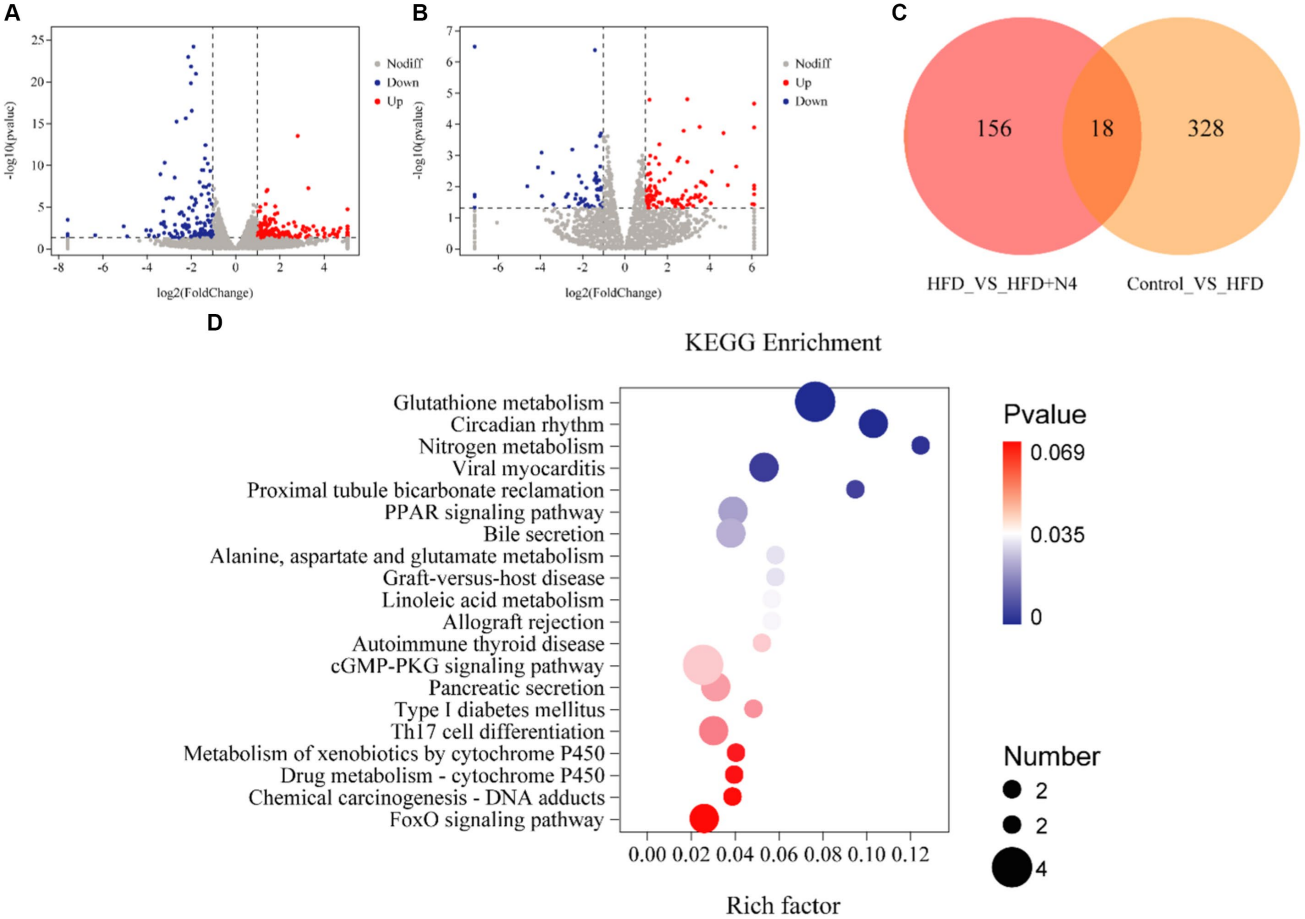
Effect of N4 intervention on transcriptome of rat liver. Volcano map of differentially expressed genes **(A)** Control vs. HFD **(B)** HFD vs. HFD + N4. Red represents up-regulated genes, blue down-regulated genes, and gray represents no difference. **(C)** Venn diagram. **(D)** Top 20 KEGG pathways of DEG in liver between HFD and HFD + N4 groups. The higher the Rich factor, the higher the enrichment. Data are shown as mean ± SD (n = 6/group). Control, normal-fat diet group; HFD, high-fat diet group; HFD + Ator, high-fat diet group treated with atorvastatin calcium tablet; HFD + N4, high-fat diet group treated with *Lpb. plantarum N4.*

The liver serves as the principal site for lipid metabolism in the body. To explore the impact of N4 on the livers of hyperlipidemic rats, we conducted transcriptome analysis on liver samples collected from each treatment group (Figures 4A,B). In a comparison of Control and HFD groups, 194 up-regulated genes and 134 down-regulated genes were identified, suggesting that the high-fat diet significantly disrupted hepatic lipid metabolism in rats. Notably, after *Lpb. plantarum* N4 intervention, 101 genes were found to be up-regulated and 55 genes were down-regulated. This suggests that N4 intervention significantly altered lipid metabolism in the liver of hyperemic rats. A total of 18 differential genes were found in both groups (Figure 4C).

During our KEGG pathway enrichment analysis, we identified 156 genes that displayed notable variances between the HFD and N4 groups, effectively linked to a comprehensive set of 171 metabolic pathways. Figure 4D provides a visual representation of the top 20 KEGG pathways associated with DEGs in the liver of both HFD and HFD + N4 groups. Notably, the pathways related to lipid metabolism included the glutathione metabolism pathway, PPAR signaling pathway, and bile secretion. Among these pathways, the glutathione metabolic pathway was the most profoundly affected and significant. In addition, *Lpb. plantarum* N4 was effective in affecting the expression levels of L-glutamic acid, glutamylspermidine and ornithine in the glutathione metabolic pathway (Supplementary Figure S5).

**Figure 5:**
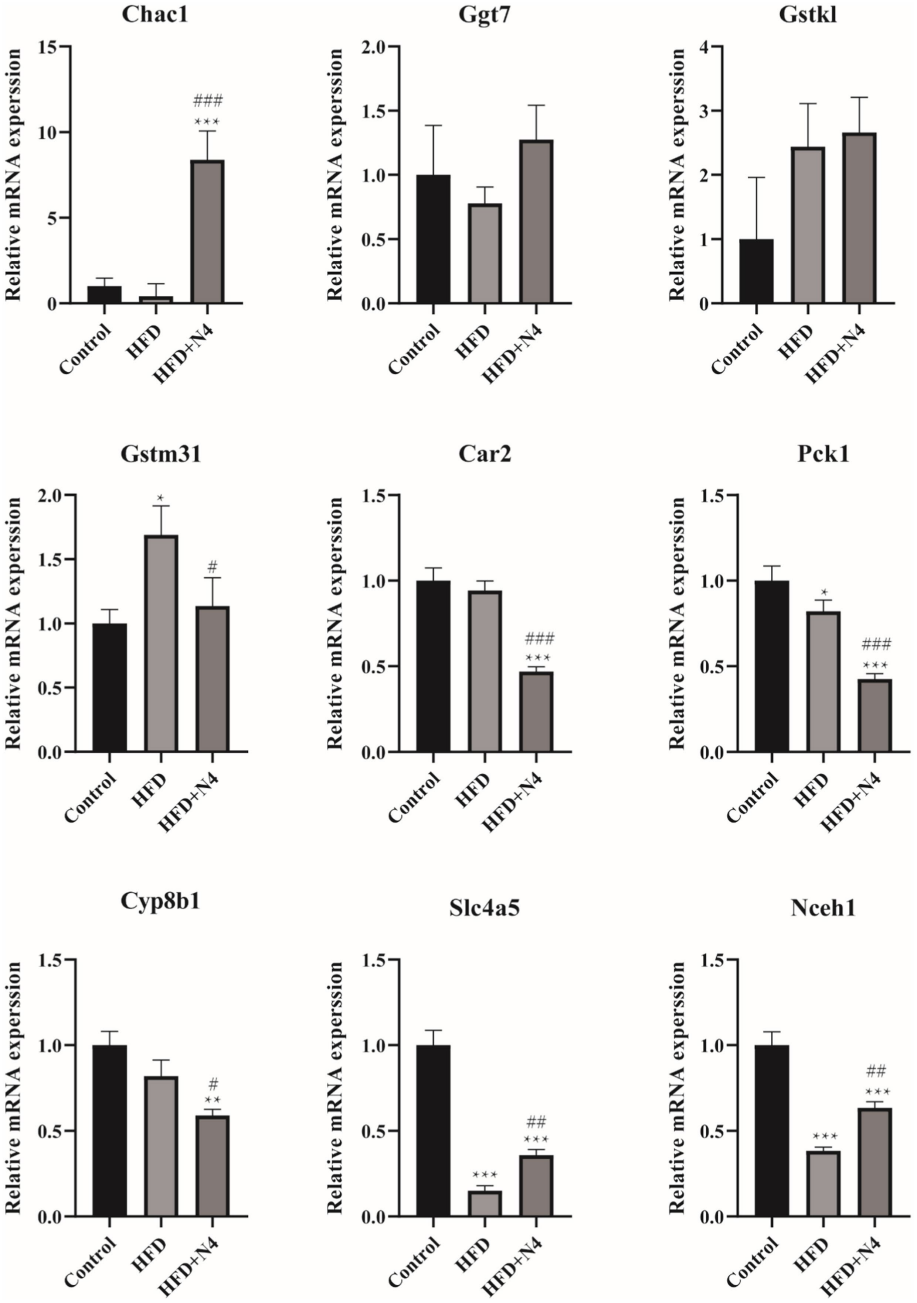
The mRNA levels were determined using real-time quantitative PCR and normalized to the β-actin mRNA expression. The results are presented as mean ± SD (*n* = 3). *p < 0.05, **p < 0.01, and ***p < 0.001 as compared with the Control group. ^#^*p* < 0.05, ^##^*p* < 0.01, and ^###^*p* < 0.001 as compared with the HFD group. Control, normal-fat diet group; HFD, high-fat diet group; HFD + N4, high-fat diet group treated with *Lpb. plantarum N4.*

To validate the gene expression profiles generated by RNA-seq analysis, we used RT-qPCR to assess the expression levels of nine genes with differential control of expression, as outlined in Supplementary Table S2. Figure 5 illustrates that the administration of N4 resulted in an increase in the mRNA levels of Chac1, Gstk1, Slc4a5, and Nceh1 (*p* < 0.001), while the expression of Car2, Pck1, and Cyp8b1 was significantly decreased (*p* < 0.05). These results are consistent with the transcriptome data.

### 3.9 Correlation analysis

**Figure 6:**
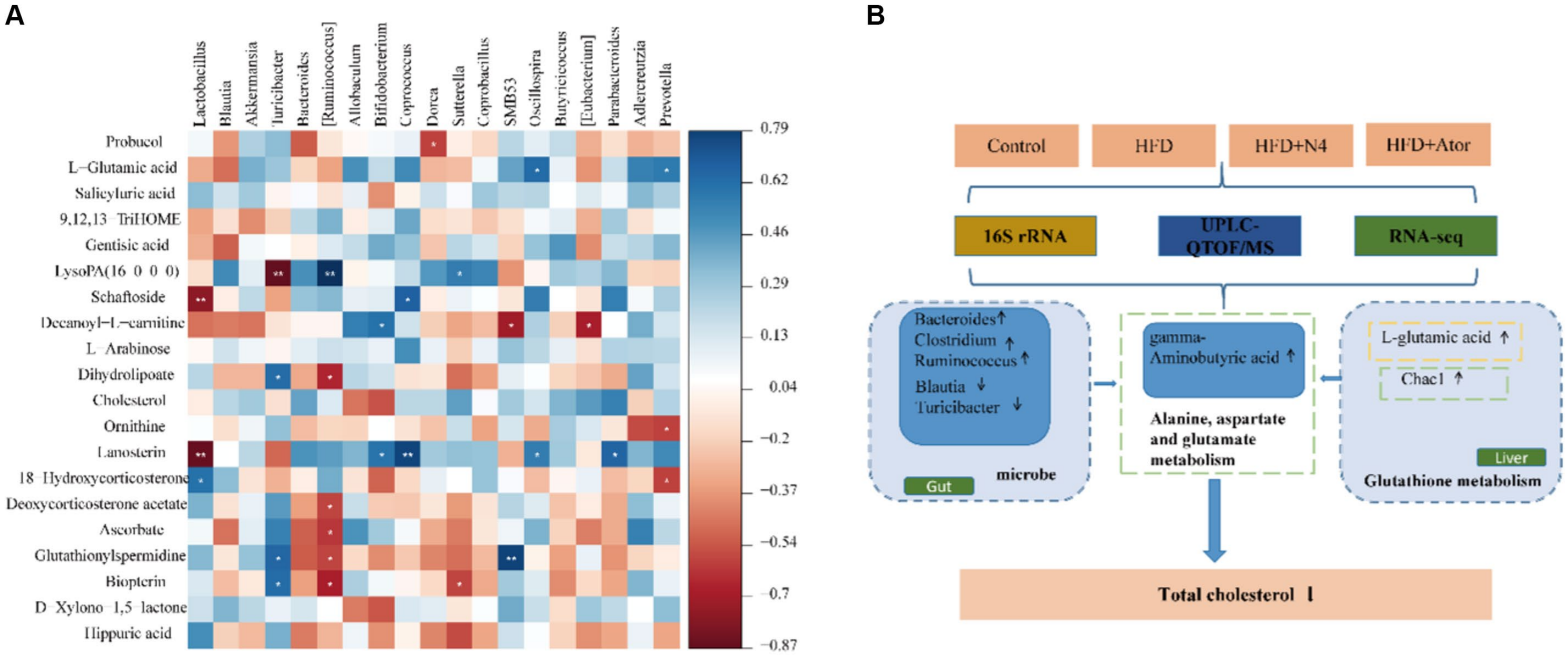
Correlation analysis. **(A)** Spearman’s correlation map of the metabolites and differential intestinal microbial community. The R values are denoted with graduated colors, and blue and red grids indicate positive and negative correlations, respectively. ***p < 0.001; ** *p* < 0.01; * *p* < 0.05. **(B)** *Lpb. plantarum N4* increases GABA as well as glutathione metabolism to regulates lipid metabolism.

To investigate the potential connection between *Lpb. plantarum* N4 and the metabolite of hyperlipidemic rats, as well as their intestinal microbiota and regulatory genes, an extensive correlation analysis was conducted using the Spearman rank correlation coefficient (Figure 6A). In the correlation analysis, values of −1 < *r* < 0 signify a negative correlation, depicted in red; while 0 < *r* < 1 denotes a positive correlation, displayed in blue; *r* = 0 indicates no correlation, represented in white. The varying shades of color reflect the strength of the correlation between gut flora and differential metabolites. Our study findings revealed a robust correlation between the quantities of *Oscillospira* and *Prevotella* microorganisms and L-glutamic acid (*p* < 0.05). Additionally, *Prevotella* was negatively correlated with ornithine levels (*p* < 0.05). *Ruminococcus* exhibited a negative correlation with glutamylspermidine levels, whereas *Bacteroides* showed a positive correlation with them (*p* < 0.05).

## 4 Discussion

In this research, five strains of lactic acid bacteria were meticulously chosen from naturally fermented na dish. Among these strains, *Lpb. plantarum* N4 demonstrated the most notable *in vitro* cholesterol-reducing capacity, remarkable resistance to acidity, and exceptional tolerance to pepsin, as well as strong viability, even in the presence of 0.3% w/v bile salts. Similar findings regarding lactic acid bacteria with such attributes have been documented previously (Edith Marius et al., 2018). Furthermore, strain N4 exhibited enhanced activity of bile brine hydrolytic enzymes while avoiding deleterious hemolytic activity or demonstrating resistance to clinically significant antibiotics. These results indicated that N4 possesses excellent probiotic properties.

The selected dosage of 5 × 10^9^ CFU/mL given to the rats in the study was not a coincidence, as many other studies have utilized similar dosages to investigate the effects of probiotics on the host. For example, a study utilized a commercial probiotic containing 3 strains of bifidobacteria and 6 strains of lactobacilli and administered it via gavage to rats at a dosage of 5 × 10^9^ CFU/mL, resulting in a significant alleviation of altitude-induced cardiac hypertrophy (Hu et al., 2022). Another study administered *Lactobacillus plantarum* 9-41-A and *Lactobacillus fermentum* M1-16 to rats via gavage at a dosage of 2 × 10^9^ CFU/mL, leading to a reduction of their total cholesterol and triglyceride levels (Xie et al., 2011). Similarly, in a clinical study, 92 participants with hypertriglyceridemia but without diabetes consumed a powder containing 5 × 10^9^ CFU/mL of *L. curvatus* HY7601 and 5 × 10^9^ CFU/mL of *L. plantarum* KY1032 each day, resulting in a significant decrease in their triglyceride levels (Ahn et al., 2015). Therefore, the dosage of 5 × 10^9^ CFU/mL has been widely utilized in the field of probiotic preparations.

Atherosclerotic cardiovascular disease is associated with high serum LDL-C and HDL-C levels (Liao et al., 2022). As expected, consumption of a high-fat diet resulted in a significant increase in lipid levels in the bloodstream. Interestingly, some probiotics are effective in reducing lipid concentrations and lipid deposition in rats fed with a high-fat diet. Previous studies have confirmed the efficacy of reducing lipids in rats fed high-fat diets using *L. rhamnosus* and *L. fermentans* (Yadav et al., 2018; Thakkar et al., 2020; Zafar et al., 2022). In our investigation, the inclusion of N4 exhibited remarkable efficacy, resulting in a 68.75% reduction in serum TC, 77.32% decrease in TG, and 67.04% decline in LDL-C. Additionally, it increased HDL-C levels by 42.03%, restoring levels to those observed in the control group. Furthermore, the application of *Lpb. plantarum* N4 led to a significant reduction in weight gain, alleviated excessive lipid droplet accumulation, and reduced the infiltration of inflammatory cells in the livers of rats fed a high-fat diet. Additionally, it demonstrated a remarkable capacity to restore both the external morphology and the internal structural integrity of the hepatic tissue. It was found that obese mice fed a high-fat diet presented a significant increase in body weight and fat accumulation, but after treatment, these changes were significantly improved, closely associated with the reconstitution of beige fat (Ma et al., 2023). Combined with our experimental results, we hypothesize that the intervention of N4 to reduce the excessive accumulation of liver fat in hyperlipidemic rats may be associated with the browning of adipocytes.

A growing body of evidence strongly supports the possibility of targeting the gastrointestinal microbiome to prevent and treat dyslipidemia (Zhang et al., 2020; Lv et al., 2021; Liu et al., 2022). Significant alterations in the composition of the gut microbiota in rats with high-fat diet-induced hyperlipidemia were observed during our investigation following the introduction of N4. These alterations were characterized by an increase in the abundance of *Sutterella*, *Bacteroides*, *Clostridium*, and *Ruminococcus*, along with a decrease in the levels of *Lactobacillus*, *Blautia*, and *Turicibacter*. *Sutterella*, a Gram-negative bacterium, is intricately involved in bile acid metabolism, thereby regulating disorders of lipid metabolism (Park et al., 2022). *Bacteroides* play a critical role in modulating gut bacteria and metabolizing tricarboxylic acid, resulting in improved liver and kidney function as well as reduced hyperlipidemia, oxidative stress, and inflammatory responses (Xie et al., 2021). *Bacteroides* has also been shown to regulate intestinal microbiota and promote the conversion of white to beige fat, reducing lipoatrophy (Wang et al., 2023). Furthermore, certain bacterial clusters, such as *Clostridium* and *Ruminococcus* have been associated with the production of SCFAs (Granado-Serrano et al., 2022). Previous studies have confirmed that a reduction in the number of *Ruminococcus* and *Clostridium* could potentially impact the synthesis of antimicrobial peptides. As a result, this can lead to the colonization of detrimental microorganisms in the intestinal barrier, consequently contributing to the progression of liver disease (Domingo et al., 2009). Notably, we observed a decrease in *Lactobacillus* abundance in rats following supplementation with *L. plantarum* N4. Distinct differences in the composition of microbial communities were observed among the different segments of the gut. To fully comprehend its significance, it is crucial to acknowledge that relying solely on high-throughput sequencing to analyze the makeup of gut bacteria is insufficient to conclusively determine whether *Lactobacillus* can effectively colonize the intestines and exert its beneficial effects (Valenzuela et al., 2018). Empirical evidence from previous studies indicates that the inclusion of *L. plantarum* does not have a significant impact on the *Lactobacillus* ratio (Chen et al., 2020). Hence, future investigations should aim to elucidate whether lactic acid bacteria can successfully establish residences in the gut. To achieve this, extensive examination of the microbiota in various regions of the gastrointestinal tract will be conducted using state-of-the-art sequencing methods. Additionally, the abundance of *L. plantarum* was quantitatively assessed using qPCR. Furthermore, it is worth noting that *Blautia* and *Turicibacter* have been implicated in lipid metabolism disorders stemming from obesity and have exhibited notable anti-obesity properties (Oba et al., 2023). *Blautia* has been found to decrease the elevated cholesterol levels caused by a high-fat diet in a mouse model with high cholesterol levels by regulating the metabolism of bile acids (Duan et al., 2021). In addition, research indicates that *Blautia* stimulates the development of beige adipocytes in WAT, regulating lipid metabolism, mitigating inflammation, and enhancing intestinal barrier function (Wang et al., 2020a). Zheng et al. revealed a direct association between *Turicibacter* and HDL-C levels (Zheng et al., 2020). The above results suggest that *Lpb. plantarum* N4 treatment can inhibit hyperlipidemia by influencing the composition of the gut microbiota.

Metabolomic analysis revealed significant disruptions in the intestinal metabolites of rats with hyperlipidemia, while the addition of *Lpb. plantarum* N4 demonstrated its effectiveness in regulating various metabolic pathways and distinct metabolites associated with hyperlipidemia. Particularly noteworthy most altered metabolites include benzene and its derivatives, carboxylic acids and their derivatives, prenol lipids, and steroids and their derivatives. KEGG pathway analysis indicated that carboxylic acids and their derivatives are strongly linked to metabolic pathways related to lipid metabolism. These pathways include neuroactive ligand-receptor interactions, sphingolipid signaling, tyrosine metabolism, cAMP signaling, sphingolipid metabolism, alanine, aspartate, and glutamate metabolism, and sterol biosynthesis. Carboxylic acids and their derivatives encompass a broad array of compounds generated through the substitution of the hydroxyl group in the carboxylic acid molecule by diverse chemical groups. These derivatives include acyl halides, anhydrides, esters, and amides, each representing distinct chemical functionalities and properties. Fumaric acid, a differential metabolite within the alanine, aspartate, and glutamate metabolic pathways, is interconnected with GABA, both of which fall under the category of carboxylic acids and their derivatives. After supplementation with *Lpb. plantarum* N4, the levels of fumaric acid decreased, whereas GABA increased. The citric acid cycle relies heavily on fumaric acid, and a decrease in its concentration suggests a compromised cycle (Yan et al., 2023). Previous studies have shown that increased fumaric acid levels in HFD-fed mice lead to cell damage and metabolic disorders (Zhou et al., 2019). Conversely, the decrease in Fumaric acid content post-N4 supplementation alleviated this situation, which is consistent with the anti-obesity effects observed in Fu’s study on alfalfa (Fu et al., 2022). GABA, classified as a non-protein amino acid, demonstrates the potential to ameliorate dysregulation within the intestinal microbiota induced by a high-fat diet, along with mitigating disorders in lipid metabolism. Research has indicated that the presence of GABA can significantly reduce both serum and hepatic TG levels in animals fed a high-fat diet, promoted hepatic lipolysis and β-oxidation, improved ecological dysregulation of the intestinal microbiota. This led to an increase in the percentage of *Bacteroidetes* and a decrease in the abundance of *Firmicutes* and *Ruminococcus* (Ma et al., 2023). Furthermore, GABA-rich fermented extracts and oolong tea have been shown to lower plasma triglyceride and low-density lipoprotein levels, down-regulate genes related to adipogenesis (SREBP-1c, FAS, SCD-1, and ACC), and promote lipid metabolism and fatty acid oxidation (Park et al., 2020; Weerawatanakorn et al., 2023). Noteworthy is the consistent finding across multiple studies that GABA induces adipocyte browning, facilitates beige fat differentiation, and acts as an inhibitor of obesity and associated metabolic disorders (Manigandan and Yun, 2022). Treatment with GABA has been shown to alter the composition of gut microbiota, elevate beige fat content, and ultimately improve metabolic levels in obese mice (Ma et al., 2023). Consequently, we posit that the elevation of GABA levels following N4 supplementation may mitigate lipid accumulation by fostering beige fat differentiation. Based on these results, we suggest that *Lpb. plantarum* N4 can regulate the metabolism of alanine, aspartate, and glutamate by influencing the concentrations of fumaric acid and GABA, and modulate the host’s digestive system to prevent the occurrence of hyperlipidemia.

Numerous scientific investigations have substantiated a robust correlation between hyperlipidemia and oxidative stress, with oxidative stress assuming a pivotal role in the development of metabolic disorders (Le Lay et al., 2014; Okuno et al., 2018; Raut and Khullar, 2023). Hyperlipidemia can readily induce oxidation of elevated levels of TC, TG, and LDL in the bloodstream. Following oxidation, these compounds permeate the blood vessel walls, inciting lipid peroxidation and generating reactive oxygen species. Consequently, the mitochondrial function is compromised, precipitating oxidative stress (An et al., 2022). Transcriptomic analysis, as revealed by KEGG enrichment, revealed pathways intricately associated with lipid metabolism, most notably the glutathione metabolic pathway, PPAR signaling, and bile secretion. Among these, the glutathione metabolic pathway was the most salient. Genes responsible for glutathione production, such as Chac1, Ggt7, and Gstk1, exhibited heightened activity, whereas genes implicated in metabolism, including Slc4a5, Car2, Pck1, and Cyp8b1, showed decreased activity. Chac1, a glutathione-specific gamma-glutamyl cyclotransferase 1, is intricately linked to glutathione. Its upregulation can ameliorate the oxidative stress resulting from glutathione depletion (Ge et al., 2022; Liu et al., 2023). Similarly, Ggt7, which is responsible for the activity of gamma-glutamyltransferase 7, plays a pivotal role in curtailing an organism’s inflammatory response (Wang et al., 2020b). GSTK 1, also known as glutathione transferase κ1, plays pivotal roles in detoxification, antioxidation, energy production, and lipid metabolism, synergistically amplifying the antioxidant processes of glutathione (Hu and Sun, 2017). Glutathione, an indispensable component of the body’s defense against deleterious oxidants, facilitates the scavenging of free radicals, thereby mitigating oxidative damage (Yu et al., 2022). Previously, an edible therapeutic fungus was harnessed to treat mice subjected to a high-fat diet, resulting in reductions in the levels of alanine aminotransferase and aspartate aminotransferase in both the liver and blood, concomitant with alterations in the prevalence of microbial populations in murine subjects. By mitigating oxidative stress and inflammation through Nrf2/NF-κB signaling, this intervention effectively resulted in hyperlipidemia (Zhang et al., 2022). These results substantiate the active involvement of the N4 variant in oxidative stress via the glutathione metabolic pathway, exerting an influence on fat storage in the host and shielding it from the adverse repercussions of aberrant lipid metabolism. Furthermore, three distinctive differential metabolites, L-glutamic acid, glutamylspermidine, and ornithine, undergo noteworthy alterations during glutathione metabolism. Notably, l-glutamic acid, a constituent of carboxylic acid and its derivatives, serves as a fundamental building block of glutathione, displaying a discernible positive correlation with the intricate domain of glutathione metabolism. Conversely, glutamylspermidine, which represents glutathione, showed a negative correlation. In contrast, ornithine exhibited an inverse association with glutathione levels, consistent with previous studies indicating that elevated ornithine levels correspond to reduced glutathione levels (Zhang et al., 2023a). Crucially, glutathione plays a pivotal role in governing lipid metabolism by inhibiting impaired fatty acid metabolism, counteracting lipid peroxidation, and reducing lipid storage concomitant with fatty acid metabolism (Miess et al., 2018; Qiu et al., 2020). In our study, the presence of *Lpb. plantarum* N4 enhanced glutathione synthesis and modulated its metabolism. This has the potential to ameliorate hepatic damage in the host and exert an influence on the host’s internal metabolic processes, ultimately leading to a reduction in fat accumulation in high-fat rats. Furthermore, within the glutathione metabolic pathway, upregulation of glutamate as a metabolite can elevate GABA content, subsequently affecting the metabolism of alanine, aspartate, and glutamate.

According to the results of the analysis of the three omics, we observed that *Lpb. plantarum* N4 exerted a dual effect (Figure 6B). On the one hand, *Bacteroides*, *Clostridium*, and *Ruminococcus* exhibited an increase in abundance, whereas *Blautia*, and *Turicibacter* declined. Conversely, it reduced the levels of Fumaric acid and elevated the levels of GABA, thus affecting the metabolism of alanine, aspartate, and glutamic acid through its influence on carboxylic acids and their derivatives. On the other hand, *Lpb. plantarum* N4 affected glutathione synthesis by regulating host hepatic glutathione metabolism, increasing the mRNA level of Chac1 and affecting changes in the level of L-glutamate, and promoting GABA synthesis. This cascade of effects resulted in the downregulation of TC and served as a preventive measure against hyperlipidemia.

Additional, recent studies have highlighted the involvement of oxidative stress in the differentiation of beige fat. Elevated oxidative stress has been shown to boost UCP1 expression and the production of markers associated with beige fat (Kahoul et al., 2023). Adipocytes, for proper functioning, rely on the activity of the nuclear receptor PPARγ. Exposure to cold or adrenergic signaling enhances thermogenic cells through various pathways that synergize with PPARγ (Zhang et al., 2023b). Activation of PPARγ has been observed to induce Ucp1 expression, express beige-characterized precursor cells, and facilitate the differentiation of adipocyte subpopulations (Harms and Seale, 2013). PPARγ, a crucial member of the PPAR family, plays a pivotal role in regulating adipocyte function and metabolism. When activated by ligands, PPAR forms activated complexes that bind to target genes, regulating the transcription of these genes. Previous studies have revealed a role for SOX4 in propelling beige adipocyte-mediated adaptive thermogenesis by promoting the formation of PRDM16-PPARγ complexes (Shen et al., 2022). In our investigation, the administration of N4 activates the PPAR signaling pathway, leading to the activation of PPARγ, which in turn triggers the expression of thermogenic genes and enhances thermogenesis. This ultimately contributes to an increase in energy expenditure. Thus, we have observed that *Lpb. plantarum* N4 enhances oxidative stress via the glutathione metabolic pathway and activates the PPAR signaling pathway, thereby stimulating PPARγ to promote beige fat differentiation.

**Figure 7:**
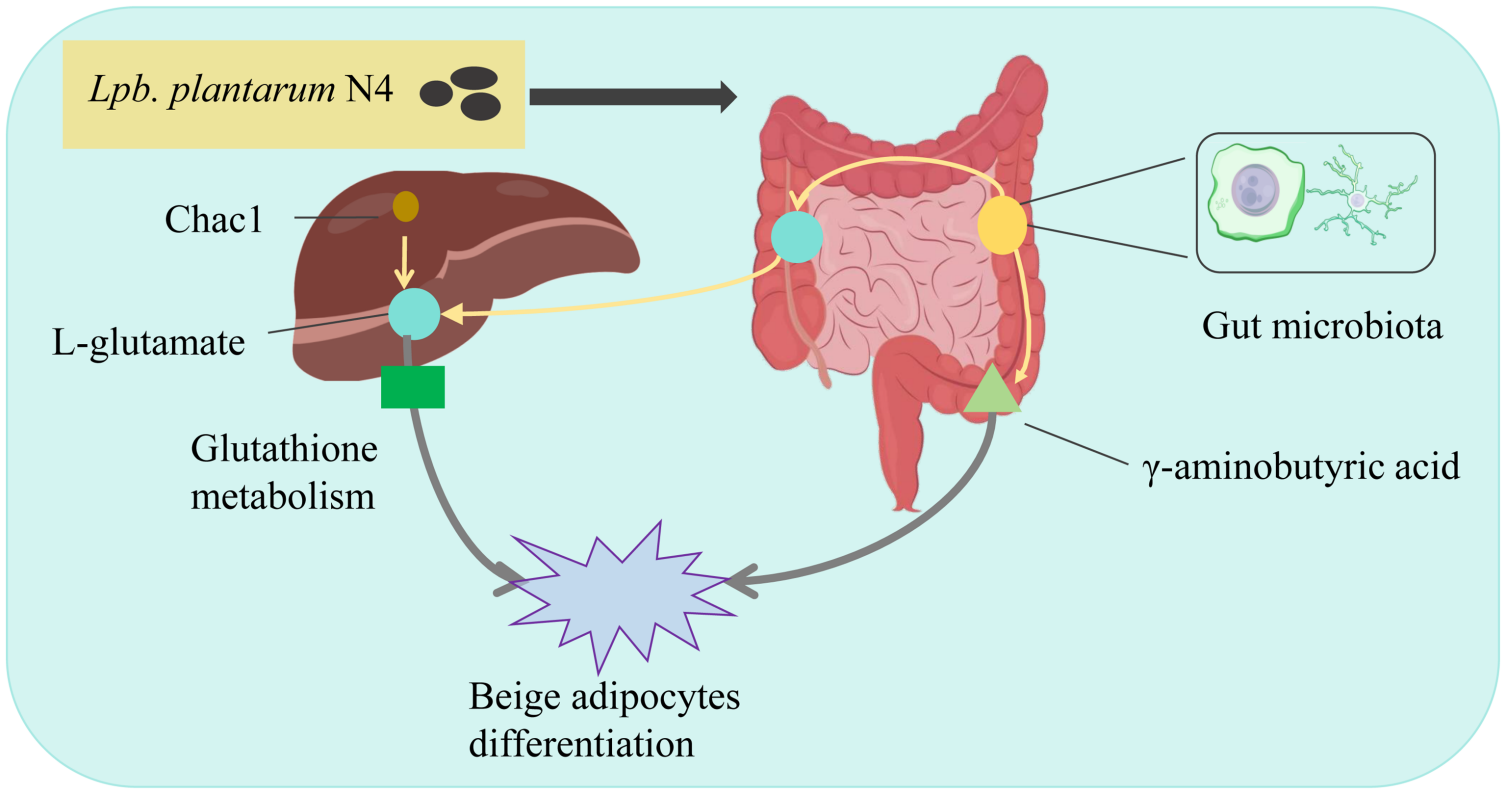
Mechanism of *Lpb. plantarum* N4 to improve lipid metabolism. N4 regulates lipid metabolism in HFD diet-induced hyperlipidemic rats by altering gut microbiota and stimulating beige lipid differentiation.

In summary, we observed a dual effect exerted by *L. plantarum* N4 (Figure 7). There was an increase in the abundance of *Bacteroides*, *Clostridium*, and *Ruminococcus*, whereas *Lactobacillus*, *Blautia*, and *Turicibacter* experienced a decline. Additionally, the heightened expression of Chac1 mRNA had an impact on L-glutamate levels, directly influencing glutathione metabolism, activating oxidative stress, and promoting GABA production, thereby affecting the beige fat differentiation pathway. Consequently, we propose that *Lpb. plantarum* N4 acts as a lipid-lowering agent within the host gut, modulating the metabolism of carboxylic acid and its derivatives through the glutathione metabolic pathway, may affect the beige fat differentiation pathway, thereby mitigating hyperlipidemia.

## 5 Conclusion

In this study, five probiotic strains exhibiting cholesterol-lowering efficacy were isolated from a traditional fermented food called na dish. Among these strains, *Lpb. plantarum* N4 demonstrated superior cholesterol-lowering efficacy and enhanced gastrointestinal fluid tolerance. Subsequent *in vivo* experiments conducted on rats with high-fat diet-induced hyperlipidemia revealed that *Lpb. plantarum* N4 supplementation improved the composition of gut microbiota and enhanced liver function. This improvement was attributed to the modulation of glutathione metabolic pathways and carboxylic acids and their derivatives, such as gamma-aminobutyric acid and L-glutamic acid, which consequently ameliorated lipid metabolism disorders in rats with high-fat diet-induced hyperlipidemia. These findings contribute significantly to our understanding of the regulatory role of *Lpb.* in lipid metabolism. However, it is worth noting that the study did not delve into whether the observed increase in gamma-aminobutyric acid following *Lpb. plantarum* N4 intervention triggered beige lipid differentiation and its impact on the gut-liver-brain axis. Further in-depth investigations are warranted to explore these aspects in subsequent studies.

## Data availability statement

The original contributions presented in the study are included in the article/Supplementary material, further inquiries can be directed to the corresponding authors.

## Ethics statement

The animal study was approved by Jiangxi Science and Technology Normal University. The study was conducted in accordance with the local legislation and institutional requirements.

## Author contributions

MD: Investigation, Methodology, Validation, Visualization, Writing – original draft, Writing – review & editing. SZ: Investigation, Visualization, Writing – original draft. SW: Investigation, Resources, Writing – original draft. QJ: Validation, Writing – original draft. WT: Writing – original draft. TL: Investigation, Writing – original draft. YO: Investigation, Writing – original draft. JL: Conceptualization, Funding acquisition, Supervision, Writing – review & editing. BG: Funding acquisition, Resources, Supervision, Writing – original draft.

## Footnotes

^1^http://blast.ncbi.nlm.nih.gov/Blast.cgi

^2^http://hmdb.ca/

^3^http://www.metaboanalyst.ca/

^4^http://hannonlab.cshl.edu/fastx-toolkit/

^5^http://www.r-project.org/
