# Supplementary material for: Lactiplantibacillus plantarum N4 ameliorates lipid metabolism and gut microbiota structure in high fat diet-fed rats
Source: Front Microbiol. 2024 Jun 7;15:1390293. doi: 10.3389/fmicb.2024.1390293 (PMC11190066; doi:10.3389/fmicb.2024.1390293)
Supplement: Supplementary file 1 [file Data_Sheet_1.pdf]

Supplementary Material

1 Supplementary Figures and Tables

1.1 Supplementary Figures

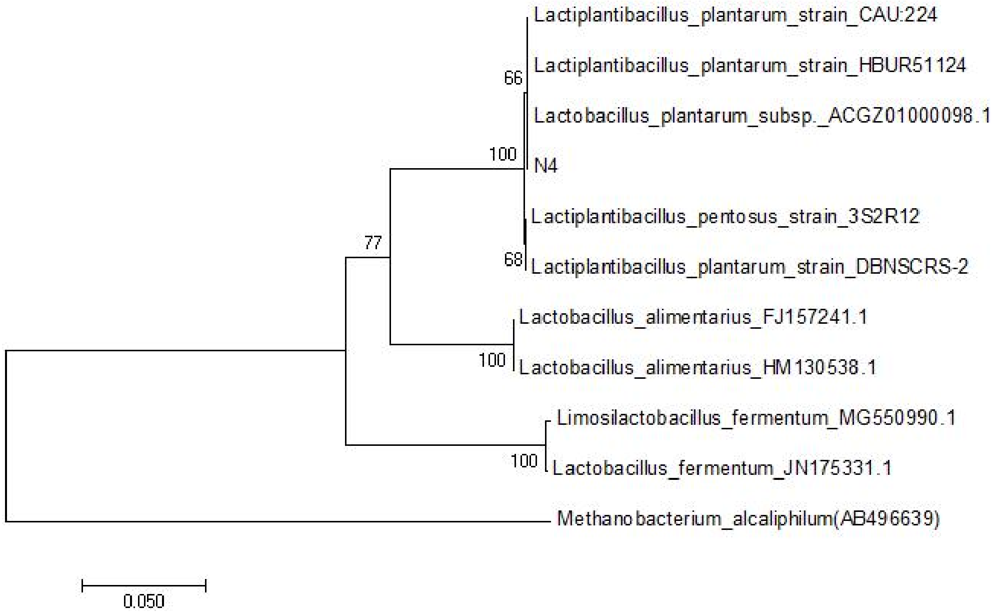

Supplementary Figure 1. Neighbor joining phylogenetic tree based on 16S rDNA sequences. Last digits are accession numbers of published sequences. Staphylococcus pasteuri strain LY-1 is the out group used for tree construction.

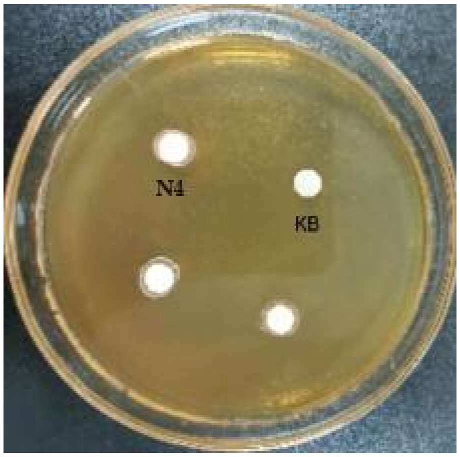

Supplementary Figure 2. Bile salt hydrolase (BSH) activity of Lactobacillus plantarum N4. KB was a blank control group, and there was no bile brine hydrolyase activity.

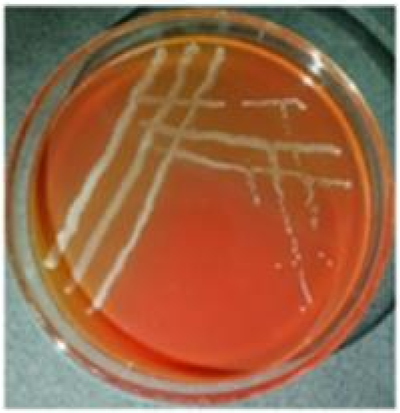

Supplementary Figure 3. Hemolytic activity test of Lactobacillus plantarum N4. No hemolytic ring.

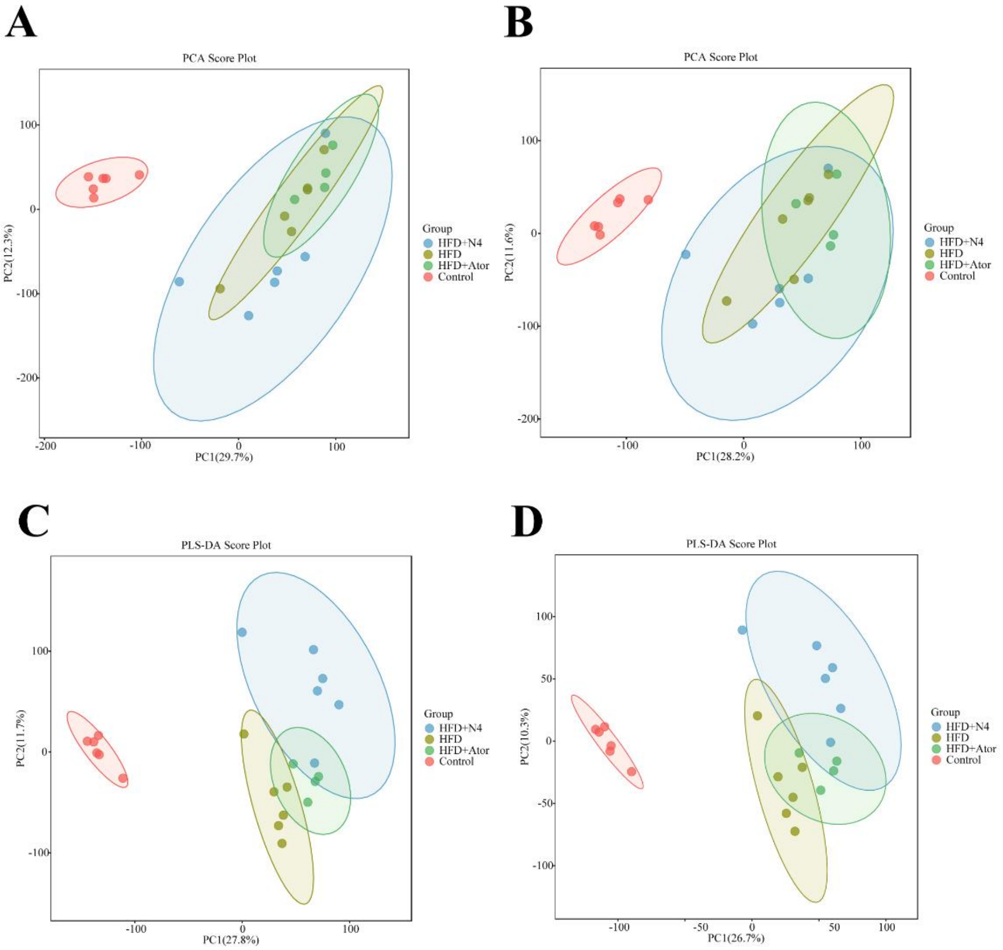

Supplementary Figure 4. Effect of N4 intervention on metabolome of rat fecal. PCA score plot (A) in the positive ion mode and (B) in the negative ion mode. PLS-DA score plot (C) in the positive ion mode and (D) in the negative ion mode. Data are shown as mean ± SD (n = 6/group). Control, normal-fat diet group; HFD, high-fat diet group; HFD+Ator, high-fat diet group treated with atorvastatin calcium tablet; HFD+N4, high-fat diet group treated with *Lpb. plantarum N4*.

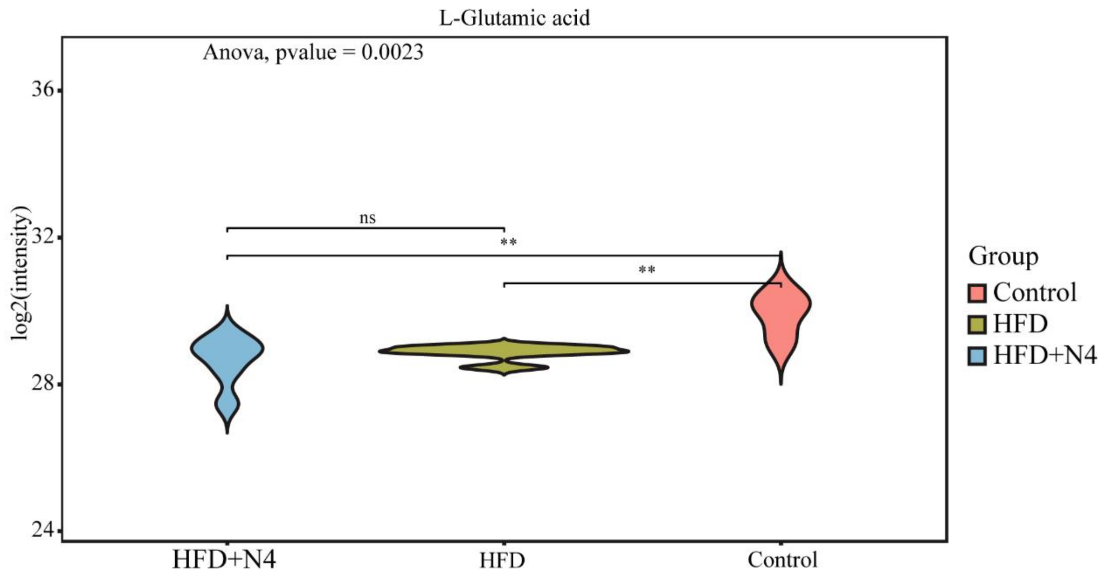
L-Glutamic acid
Anova, pvalue = 0.0023
36
32
28
24
log2(intensity)
ns
**
**
HFD+N4
HFD
Control
Group
Control
HFD
HFD+N4

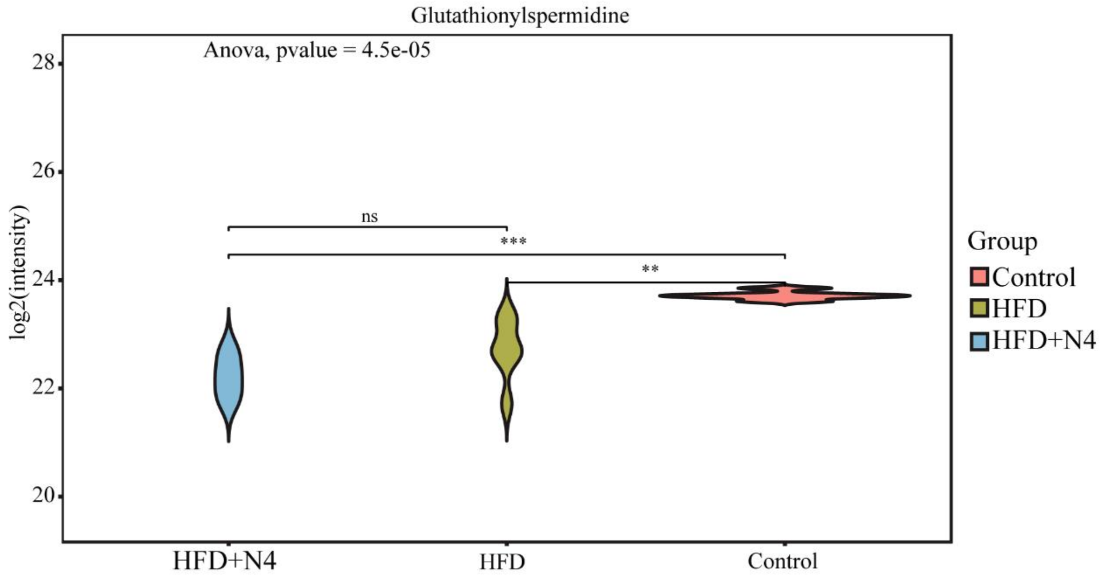
Glutathionylspermidine
Anova, pvalue = 4.5e-05
28
26
24
22
20
log2(intensity)
ns
***
**
HFD+N4
HFD
Control
Group
Control
HFD
HFD+N4

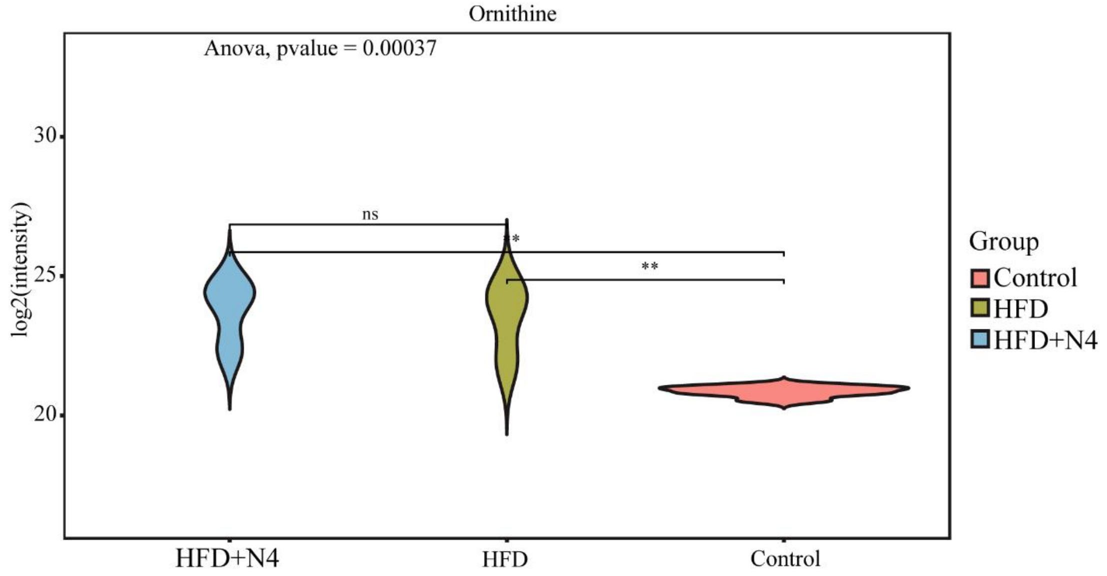
Ornithine
Anova, pvalue = 0.00037
30
25
20
log2(intensity)
ns
**
**
HFD+N4
HFD
Control
Group
Control
HFD
HFD+N4

Supplementary Figure 5. Three representative differentially expressed metabolites: L-glutamic acid, glutathionylspermidine, ornithine. *$p < 0.05$ and **$p < 0.01$. Data are shown as mean ± SD (n = 6/group). Control, normal-fat diet group; HFD, high-fat diet group; HFD+Ator, high-fat diet group treated with atorvastatin calcium tablet; HFD+N4, high-fat diet group treated with *Lpb. plantarum* N4.

2 Supplementary Tables

Table S1. Real-time quantitative PCR primer sequence of liver lipid metabolism-related genes

Gene name	Forward primer (5′−3′)	Reverse primer (5′−3′)
β-ACTIN	GACGTTGACATCCGTAAAGAC	CTAGGAGCCAGGGCAGTA
Chac1	TTTGCCTACAGCGACAGC	TTCAAGGAGGGTCACCACT
Gstm3l	TGGACACCCGCATACAT	CGCTTGCCCAGGAACT
Ggt7	TCACCGCCTGCCTC	CGTCACCACAGCACCTT
Gstk1	TGGCTGGGCTTTGAGG	CATAGCTGGTGGTTGGTTTC
Cyp8b1	TCCGCAGATTTGACCTACTTT	ACCAGTTACTTATGCCGTCTTT
Pck1	CGGATACGGTGGGAACT	TGCTGCCAGGTATTTCTTCTT
Slc4a5	CTGCTAAGAACACACAGTGATGA	GGGGACTGGAAAGCCAATA
Nceh1	ACGCCGTCATCGTTTCTAT	GGGTCCACCTTGTACTTGTCT
Car2	GTGCTCAAGGAACCCATTAC	CGCCAGTTGTCCACCAT

Table S2 Differentially expressed genes related to lipid metabolism

ID	Gene name	log2FoldChange	P
ENSRNOG00000014387	Chac1	1.465607468	0.019853167
ENSRNOG00000019058	Gstm3l	3.812004049	0.023200161
ENSRNOG00000018441	Ggt7	2.462779717	0.035571835
ENSRNOG00000016484	Gstk1	-1.302566527	0.020444156
ENSRNOG00000019481	Cyp8b1	1.768930945	0.033208979
ENSRNOG00000028616	Pck1	1.514495081	0.047194573
ENSRNOG00000010378	Slc4a5	2.964953415	0.040288093
ENSRNOG00000013313	Nceh1	1.118121677	0.048450513
ENSRNOG00000009629	Car2	-1.254967137	0.022350204
